# Combined bezafibrate, medroxyprogesterone acetate and valproic acid treatment inhibits osteosarcoma cell growth without adversely affecting normal mesenchymal stem cells

**DOI:** 10.1042/BSR20202505

**Published:** 2021-01-05

**Authors:** Jonathan J. Sheard, Andrew D. Southam, Hannah L. MacKay, Max A. Ellington, Martyn D. Snow, Farhat L. Khanim, Christopher M. Bunce, William E. Johnson

**Affiliations:** 1 Life and Health Sciences, Aston University, Birmingham B4 7ET, U.K.; 2 School of Biosciences, University of Birmingham, Birmingham B15 2TT, U.K.; 3 Institute of Cancer and Genomic Studies, University of Birmingham, Birmingham B15 2TT, U.K.; 4 University Centre Shrewsbury, Guildhall, Frankwell Quay, Shrewsbury SY3 8HQ, U.K.; 5 Royal Orthopaedic Hospital, Birmingham B31 2AP, U.K.; 6 School of Biomedical Sciences, University of Birmingham, Birmingham B15 2TT, U.K.; 7 Chester Medical School, Faculty of Medicine and Life Sciences, University of Chester, Chester CH1 4BJ, U.K.

**Keywords:** cell death, cell proliferation, drug repurposing, mesenchymal stem cell, osteosarcoma

## Abstract

Drug repurposing is a cost-effective means of targeting new therapies for cancer. We have examined the effects of the repurposed drugs, bezafibrate, medroxyprogesterone acetate and valproic acid on human osteosarcoma cells, i.e., SAOS2 and MG63 compared with their normal cell counterparts, i.e. mesenchymal stem/stromal cells (MSCs). Cell growth, viability and migration were measured by biochemical assay and live cell imaging, whilst levels of lipid-synthesising enzymes were measured by immunoblotting cell extracts. These drug treatments inhibited the growth and survival of SAOS2 and MG63 cells most effectively when used in combination (termed V-BAP). In contrast, V-BAP treated MSCs remained viable with only moderately reduced cell proliferation. V-BAP treatment also inhibited migratory cell phenotypes. MG63 and SAOS2 cells expressed much greater levels of fatty acid synthase and stearoyl CoA desaturase 1 than MSCs, but these elevated enzyme levels significantly decreased in the V-BAP treated osteosarcoma cells prior to cell death. Hence, we have identified a repurposed drug combination that selectively inhibits the growth and survival of human osteosarcoma cells in association with altered lipid metabolism without adversely affecting their non-transformed cell counterparts.

## Introduction

Combinatorial drug treatments are more effective in the treatment of many different types of tumour than monotherapies as this increases the number of aberrant signalling or pathogenic pathways that can be targeted. In osteosarcoma, which, although rare, is the most common form of bone tumour affecting children and adolescents [1], the current chemotherapeutic drug regimen includes a combination of methotrexate, cisplatin, doxorubicin and ifosfamide [2]. However, these drugs demonstrate toxicity against normal cells and have limited effect on metastatic osteosarcoma, for which patients have a relatively poor prognosis [2,3]. Therefore, new drug combinations are required that selectively target osteosarcoma and do not adversely affect normal cell counterparts. This search for new anti-tumour therapies has included a strategic repurposing of drugs that are already licensed for clinical application and of proven safety [4]. For example, the histone deacetylase inhibitor, valproic acid (VPA), which is used clinically to treat epilepsy [5], augments the effects of cytotoxic anti-tumour drugs against osteosarcomas by enhancing expression of apoptosis-related genes [6].

In blood cancers, i.e. leukaemia and lymphoma, combination therapies and drug repurposing, as well as problems of drug toxicity, are also seen as strategically important and novel drug regimens have successfully increased patient survival rates for some forms of haemopoietic malignancy. For example, we recently combined the lipid-regulating drug bezafibrate (BEZ) and the sex hormone medroxyprogesterone acetate (MPA) (combined treatments were termed BaP) to induce cell growth arrest, apoptosis and increased differentiation of acute myeloid leukaemia (AML) *in vitro* [7]. In addition, we showed therapeutic efficacy in a clinical trial of low dose BaP therapy in elderly and relapsed AML [8]. BaP therapy also prevented disease progression in a phase I/II trial in endemic Burkett’s lymphoma [9]. There were no evident adverse effects on normal haemopoietic stem cells or progenitors at the low drug concentrations used in the present study. The mechanisms of action of these drugs are complex and likely to be disease-specific, although data suggest that generation of reactive oxygen species (ROS) and altered prostaglandin and fatty acid synthesis is important [8,10]. Disruption of stearoyl CoA desaturase 1 (SCD-1)-mediated lipogenesis, in particular, was associated with the cytotoxic effects of BaP against AML [10].

Aberrant signalling and metabolic pathways that BaP target are implicated in the formation of tumours thought to derive from the transformation of mesenchymal stromal/stem cells (MSCs), including osteosarcoma [11] and chondrosarcoma [12]. Hence, there is an intriguing possibility that the biochemical, molecular and cytotoxic effects of drugs that have been successfully re-profiled to treat haemopoietic malignancies may also prove efficacious in other forms of cancer, i.e. MSC-derived sarcomas. Therefore, the present study has examined the effects of BEZ, MPA and VPA, alone and in combination, on the growth, survival and migratory behaviour of human osteosarcoma cells in comparison with normal (non-transformed) human MSCs.

## Methods

### Cell culture

Human MSCs were obtained from excised human knee fat-pad tissue following donor informed consent and ethical approval (12/EE/0136; National Research Ethics Service (NRES) Committee, East of England Hertfordshire). The experiment protocol for the research involving humans and donated tissues was in accordance with NRES guidelines following ethical approval and the Declaration of Helsinki. The adipose tissue was washed and digested with collagenase and MSCs selected according to their preferential adhesion to tissue culture plastic before culture expanding in DMEM/F12 culture medium supplemented with 10% fetal calf serum (FCS) and 1% penicillin and streptomycin (standard culture medium, all Gibco, Fisher Scientific, Loughborough, U.K.) in a humidified atmosphere at 37°C and 5% CO_2_. Stock cultures of MSCs, SAOS2 and MG63 osteosarcoma cells were maintained by routine passaging at 70% confluence and re-seeding into fresh culture flasks at a density of 5 × 10^3^ cells/cm^2^. We have shown previously [13] that these adipose-derived MSCs exhibit the necessary characteristics in terms of plastic adherence, CD immunoprofile and differentiation capacity to be recognised by the International Society for Cell Therapies as MSCs [14].

### Drug treatments

BEZ, MPA and VPA (all Sigma-Aldrich Ltd., Dorset, U.K.) were stored at −20°C as stock concentrations of 10^−2^ M (in dexamethasone, ethanol or cell culture dH_2_O, respectively). After thawing, these were diluted to final concentrations as indicated in the results section in standard culture medium. Identical dilutions of solvent carrier(s) alone were used in control cultures. Human MSCs, SAOS2 or MG63 cells were seeded in standard culture medium in 48- or 96-well plates at a density of 5 × 10^3^ cells/well and left to adhere overnight. After this time, the culture medium was replaced with drug-supplemented or control culture medium and cells cultured for a further 120 h in a humidified atmosphere at 37°C and 5% CO_2_.

### Cell viability assays

Staining with tetrazolium dye 3-(4,5-dimethylthiazol-2-yl)-2,5-diphenyltetrazolium bromide (MTT; Sigma-Aldrich Ltd) and DRAQ7™ (Bio Status Ltd, Leicestershire, U.K.) was used to measure metabolically active (viable) cell numbers, adapting methods previously reported [15]. For the MTT assay, the enzymatic formation of formazan was measured by spectrophotometry at 492 nm, corrected for background values at 650 nm, and these values have been presented normalised to the mean control values. Viability was further assessed by DRAQ7™ (Abcam Ltd., Cambridge, U.K.), a fluorescent dye that stains permeabilised dead/dying cells without adversely affecting viable cells. DRAQ7™ incorporation was quantitated using the Cell-IQ system during live-cell imaging and lineage tracking, as described below.

### Live cell image capture and analysis

Live-cell imaging and analysis were conducted using the Cell-IQ Imagen live-cell imaging platform and Cell-IQ Analyser image analysis software (Cell-IQ version 2, CM Technologies Oy, Tampere, Finland), which performs continuous live-cell imaging of cells maintained within an incubator under intermittent phase contrast and fluorescence microscopy. Upon selection of desired regions of interest (ROI), the system was used to collect digitised images over time. For lineage tracking, the Cell-IQ Analyser Manual Cell Lineage-Tracking tool was used to track individual cells of each cell type throughout the culture period, i.e. for each experiment using human MSCs, SAOS2 and MG63 cells, where the mean numbers of cells examined in each experiment were 35 ± 11, 92 ± 17 and 62 ± 18 (mean ± standard error of the mean), respectively. The proliferation and migration of cells was assessed using the same collected digitised images and software, where we measured: (i) the number of viable cell divisions; (ii) the trajectory distance; (iii) the migratory speed of viable cells for human MSCs, SAOS2 and MG63 cells throughout the culture period.

### Western blotting

Immunoblotting for the lipogenic enzymes, FASN and SCD-1, was performed as described previously [10]. In brief, V-BAP treated and control sub-confluent (80%) cultures of SAOS2, MG63 cells and human MSCs were harvested after 48 h by trypsinisation and pelleted by centrifugation. Following suspension in phosphate buffered saline (PBS) and re-pelleting, cell extracts were prepared by lysis using radioimmunoprecipitation assay (RIPA) buffer. Thirty micrograms of protein for each cell sample was combined with Laemmli 4× loading dye (Bio-Rad, Fisher Scientific Ltd.) and 10% β-mercaptoethanol (Sigma-Aldrich Ltd.) and heated for 5 min at 70°C. Cell extracts were separated using SDS-PAGE precast gradient gels (4%-15%; Bio-Rad), proteins transferred to Immobilon-P membrane (Millicorp, Sigma-Aldrich Ltd.). Membranes were sectioned in two parts at ∼60 KDa, the lower half probed with 1/1000 dilutions of anti-SCD-1 (ab19862; Abcam Ltd.), and the upper half with anti-ACC1 (4190; New England Biolabs Ltd.), anti-phospho-ACC Ser79 (11818; New England Biolabs Ltd.), and anti-FASN (ab22759; Abcam Ltd). The lower half of the membrane was probed with anti-β-actin antibodies (SigmaAldrich Ltd) diluted at 1/25,000 as loading control. Densitometry was performed using ImageJ software (http://rsb.info.nih.gov/ij/) and protein expression normalized to β-actin.

### Statistical analysis

At least three independent experiments were performed for all data shown, i.e., testing the effects of single treatment and drug combinations on human MSC cultures from at least three different donors and testing the SAOS2 and MG63 cell lines on at least three independent occasions. Data were tested for normal distribution and two-way ANOVAs used to examine the significance of drug treatments on viable cell number, cell division and cell migration with post-hoc Tukey tests or *t* tests applied to examine differences between individual groups and concentrations. Data have been shown as means±standard error of the mean (SEM); *P* values of <0.05 were considered significant.

## Results

### The comparative effects of repurposed drugs on human osteosarcoma versus normal MSC growth, viability and migration

**Figure 1:**
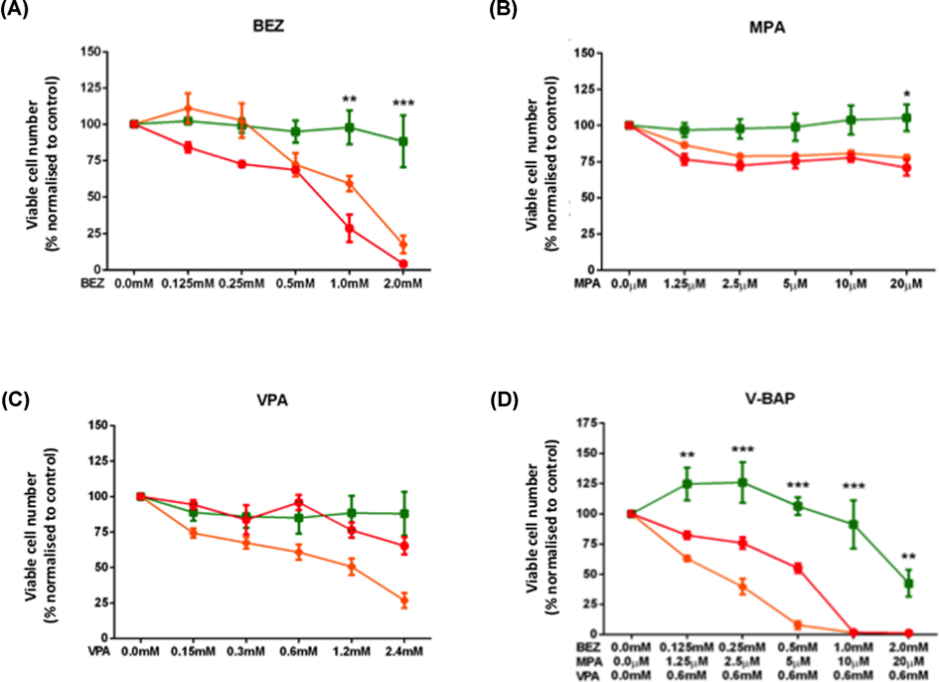
Combined treatments with V-BAP selectively inhibited SAOS2 and MG63 osteosarcoma cell proliferation MTT assays of viable cell numbers were performed after 120 h of culture in the presence of increasing concentrations of (**A**) BEZ, (**B**) MPA and (**C**) VPA as single drug treatments. Cells are designated as follows: SAOS2: orange, MG63: red, human MSCs: green. (**D**) Drug combinations of VPA (0.6 mM) with increasing concentrations BEZ and MPA (termed, V-BAP). Data shown as mean ± SEM (*n*=3 independent experiments) **P<0.01, ***P<0.001. Significant differences between both osteosarcoma cell lines and human MSCs have been indicated only.

Combined treatments with BEZ, MPA and VPA (termed V-BAP) were more effective in inhibiting the growth and survival of SAOS2 and MG63 osteosarcoma cells than single treatments of each drug alone (Figure 1). The effects on V-BAP on the numbers of viable osteosarcoma cells (assessed by MTT assay) were significant and drug concentration-dependent. Hence, at V-BAP concentrations of 1 mM BEZ, 10 μM MPA and 0.6 mM VPA, the number of viable SAOS2 and MG63 cells present in treated cultures was less than 2% of control numbers. In contrast, V-BAP treatment had little effect on the growth and survival of human MSCs, such that at a V-BAP concentration of 1 mM BEZ, 10 μM MPA and 0.6 mM VPA, the viable MSC number was still approximately 95% of control values, with no significant difference. At greater concentrations of V-BAP, i.e. at 2 mM BEZ, 20 μM MPA and 0.6 mM VPA, the number of viable human MSCs decreased to less than 50% of control values. Hence, there was an effective concentration range of V-BAP that blocked the growth of SAOS2 and MG63 cells, but had no adverse effect on human MSCs. Furthermore, in separate studies we found that V-BAP treatment was significantly more effective at inhibiting SAOS2 and MG63 cell proliferation than the drug combination of BaP alone (Supplementary Figure S2).

The combined drug treatments of 0.5 mM BEZ, 5 μM MPA and 0.6 mM VPA (designated V-BAP Low) versus 1 mM BEZ, 10 μM MPA and 0.6 mM VPA (designated V-BAP High) marked a transition from drug concentrations that prevented the growth of SAOS2 cells only to a concentration that effectively prevented the growth of SAOS2 and MG63 cells, with the growth of human MSCs remaining unaffected. Therefore, these two concentrations were examined further.

**Figure 2:**
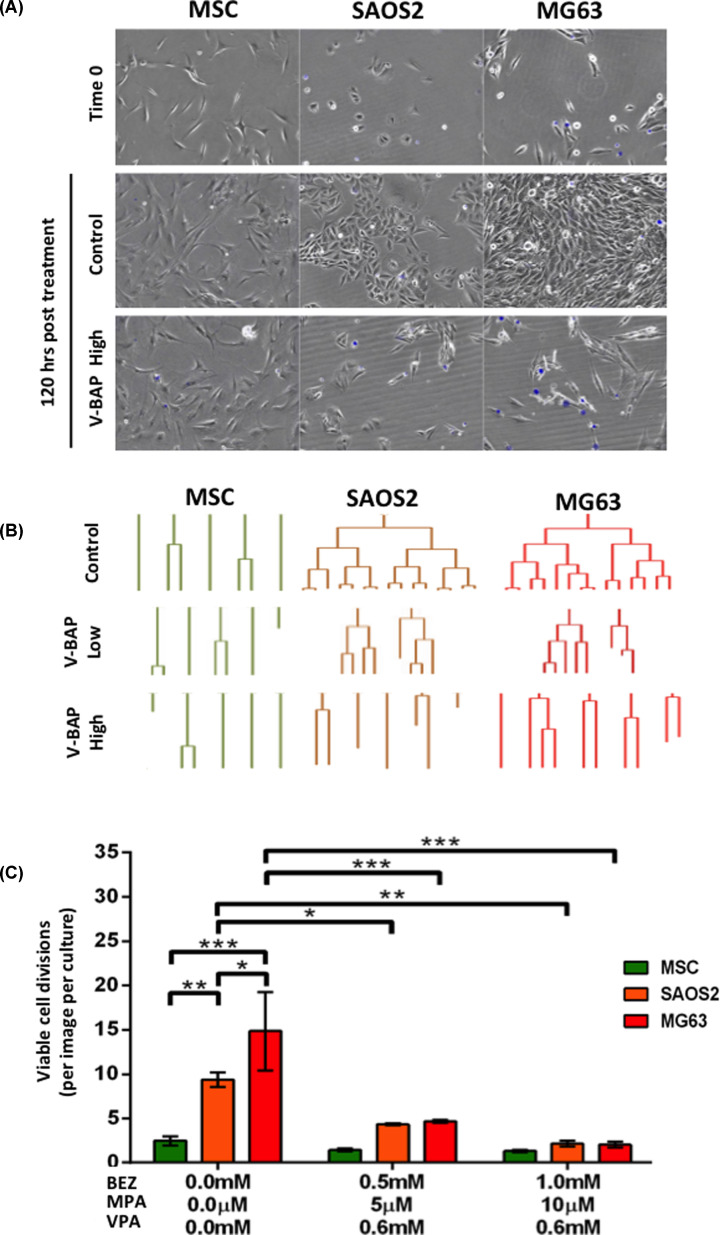
V-BAP treatment induced cell death and significantly inhibited SAOS2 and MG63 cell division with minimal effect on human MSCs (**A**) Representative digitised images of human MSCs, SAOS2 and MG63 cells within control conditions at 0 h (top panels) and at 120 h culture in control (upper middle panels) versus V-BAP High [1 mM BEZ, 10 μM MPA, 0.6 mM VPA] treated conditions (lower middle panels). Non-viable DRAQ7™ stained cells appear blue. (**B**) Representative lineage trees of human MSCs (green), SAOS2 (orange) and MG63 cells (red) tracked over 120 h of culture in control conditions (top panels) versus V-BAP Low [0.5 mM BEZ, 5 μM MPA, 0.6 mM VPA] (middle panels) and V-BAP High [1 mM BEZ, 10 μM MPA, 0.6 mM VPA] (bottom panels) treatment conditions. Lineage termination prior to the end of 120 h indicates cell death (determined by DRAQ7™ staining). (**C**) The numbers of viable cell divisions in control versus V-BAP Low and V-BAP High treatments over 120 h. Data shown as means ± SEM (*n*=3 independent experiments); *P<0.05, **P<0.01, ***P<0.001.

We used time-lapse live cell image analysis combined with DRAQ7™ staining to examine whether V-BAP treated cells were undergoing growth arrest and/or were also dying. SAOS2 and MG63 cells showed greater proliferation than their non-transformed MSC counterparts, as measured by the number of cell divisions recorded over a 120-h culture period. However, there was a significant reduction in the number of viable SAOS2 and MG63 cells undergoing cell division (versus control cultures) after drug treatments, which was associated with an increase in cells staining positively for DRAQ7 (Figure 2). In contrast, there was no significant change in the numbers of viable MSCs undergoing cell division when treated with either V-BAP Low or V-BAP High concentrations. This data confirmed that V-BAP treatment was not simply affecting cell metabolic rates, which the MTT assay would also detect, but was inhibiting osteosarcoma cell proliferation and survival.

**Figure 3:**
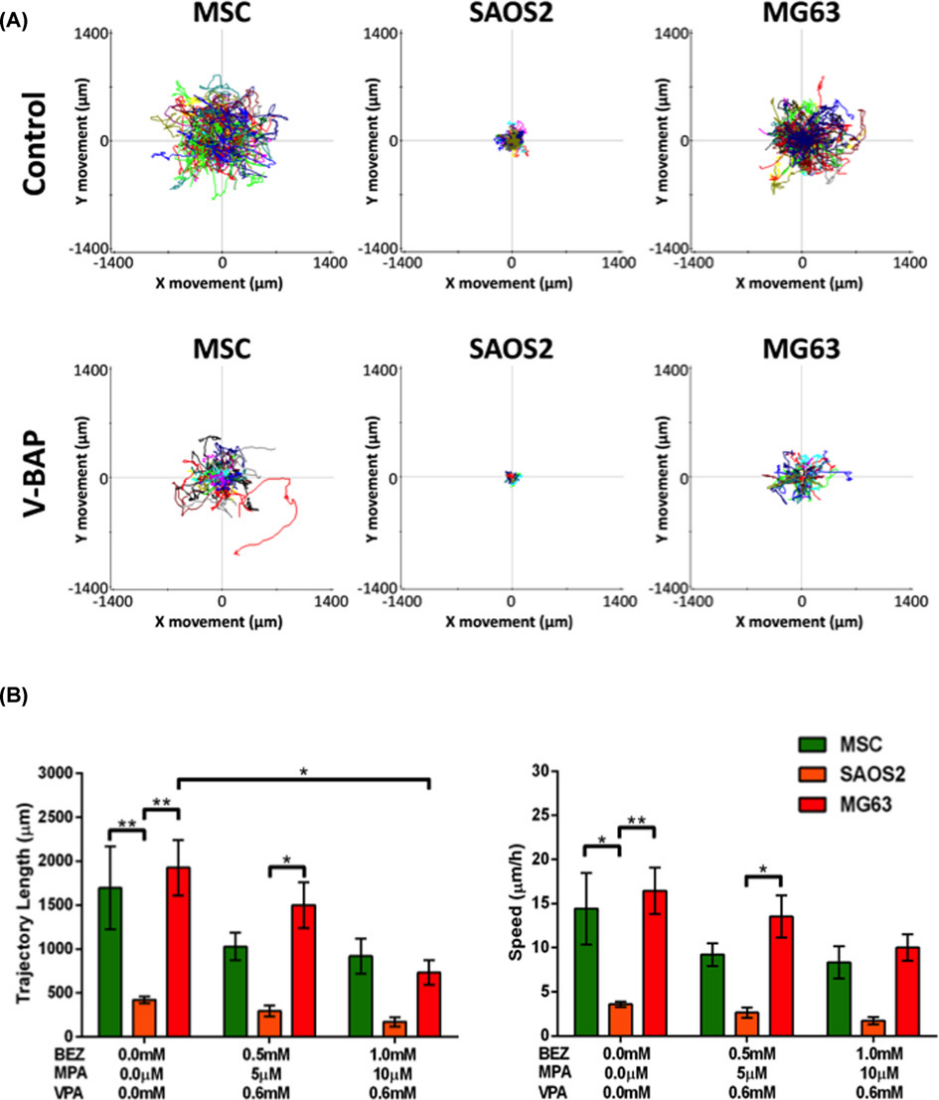
V-BAP treatment inhibited cell migration (**A**) Representative centred trajectory plots illustrate the trajectory paths of multiple human MSCs, SAOS2 and MG63 cells over 120 h of treatment in control (top panels) and V-BAP High [1 mM BEZ, 10 μM MPA, 0.6 mM VPA] (bottom panels) treatment conditions. (**B**) The trajectory lengths (left graph) and migratory speeds (right graph) of human MSCs (green), SAOS2 (orange) and MG63 (red) cells in control versus V-BAP Low [0.5 mM BEZ, 5 μM MPA, 0.6 mM VPA] and V-BAP High [1 mM BEZ, 10 μM MPA, 0.6 mM VPA] treated cultures. Data shown as means ± SEM (*n*=3 independent experiments), *P<0.05, **P<0.01.

In the absence of drug treatment, MG63 cells and human MSCs had a migratory phenotype, with increased trajectory lengths and migratory speeds when compared with SAOS2 cells (Figure 3A). However, cell migration was inhibited for all cell types following treatment with V-BAP. For MG63 cells only, the inhibitory effect of V-BAP treatment on trajectory length was significant (Figure 3B).

### The lipogenic enzymes fatty acid synthase (FASN) and stearoyl CoA desaturase (SCD-1) are elevated in human osteosarcoma cells compared with MSC and down-regulated after V-BAP treatment

**Figure 4:**
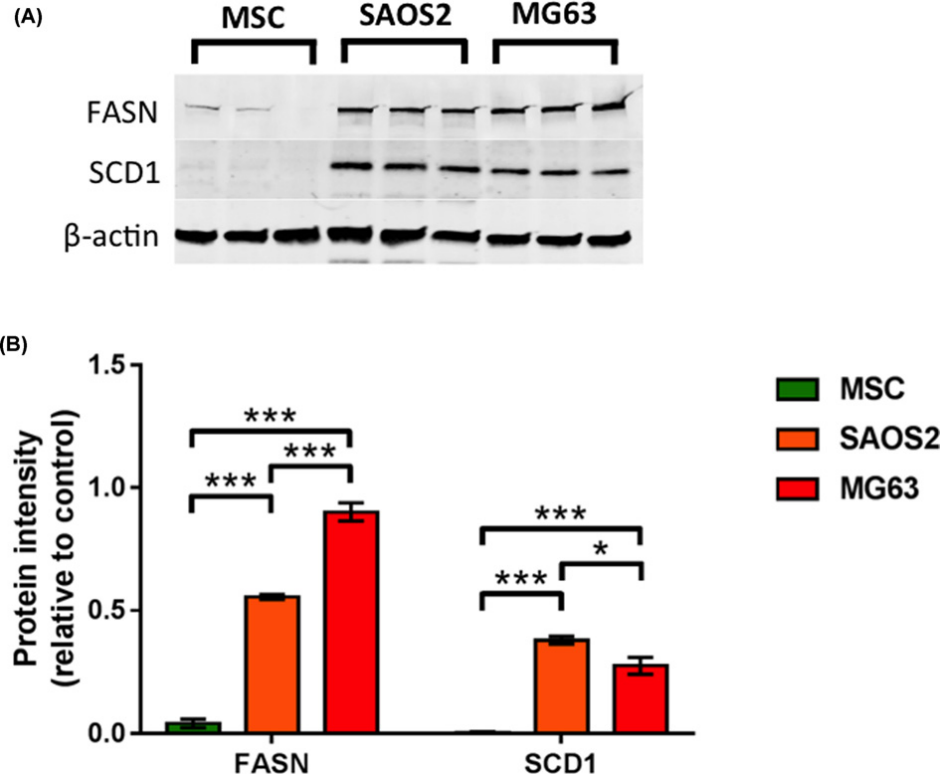
SAOS2 and MG63 osteosarcoma cells express greater levels of FASN and SCD-1 compared with human MSCs (**A**) Representative immunoblots of cell extracts for FASN and SCD-1 from human MSCs (*n*=3 donors shown), SAOS2 and MG63 cells. (**B**) Scanning densitometry of FASN and SCD-1 protein levels. Data shown as means ± SEM (*n*=3 experiments); *P<0.05, **P<0.01.

**Figure 5:**
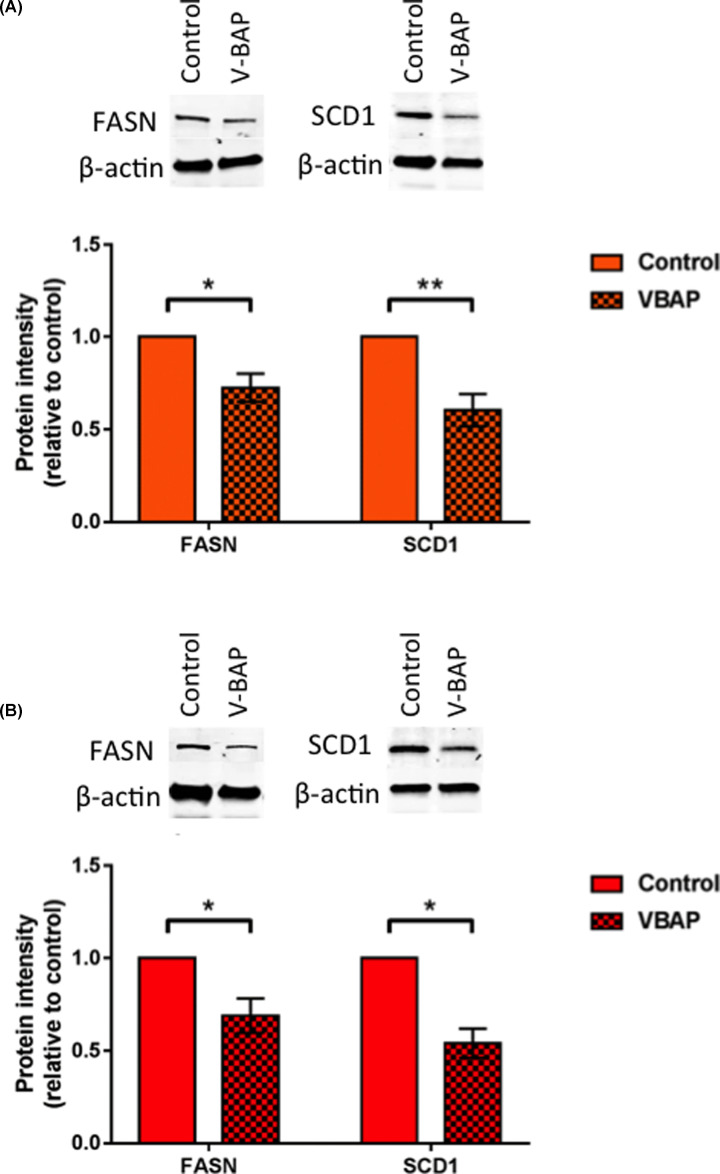
V-BAP treatment down-regulated FASN and SCD-1 levels in SAOS2 and MG63 osteosarcoma cells Cell lysates were prepared for SAOS2 (**A**) and MG63 (**B**) cells treated for 48 h with either control versus V-BAP High [1 mM BEZ, 10 μM MPA, 0.6 mM VPA]. Proteins were separated on gradient SDS-PAGE. After transfer, membranes were cut in half and the upper half probed for FASN. The lower half of the membrane was probed for SCD1 and β-actin as loading control Representative western blots are shown together with bar charts of means ± SEM of *n*>3 experiments. Significance is denoted by *P<0.05, **P<0.01.

Immunoblotting of cell extracts demonstrated that FASN and SCD-1 levels were very low or non-detectable in human MSCs compared with the osteosarcoma cell lines (Figure 4). However, following 48 h of V-BAP High treatment there was a significant decrease in FASN and SCD-1 in SAOS2 and MG63 cell lines (Figure 5). At this time point post-treatment, the V-BAP treated and control SAOS2 and MG63 culture viabilities were not significantly different and greater than 95% cells were viable in all cases.

## Discussion

A combinatorial drug regime of BEZ and MPA (BaP) was recently demonstrated to target AML and endemic Burkitt’s lymphoma with little effect on the survival of normal adult haemopoietic progenitor cells [7,9]. In addition, the histone deacetylase (HDAC) inhibitor, VPA, was reported to reduce the growth-inhibitory and cytotoxic effects of doxorubicin in osteosarcoma cell lines [16]. Therefore, we hypothesised that a similar drug regime of BEZ and MPA combined with VPA may selectively target osteosarcoma cells, while preserving their non-transformed cell counterparts, i.e. human MSCs. Following individual drug treatments for 5 days (120 h), BEZ and VPA treatments alone significantly reduced SAOS2 and MG63 cell viability to a greater extent than treatments with MPA alone, in a concentration-dependent manner. We then combined BEZ and MPA treatments in doubling dilutions that mimicked those previously reported as effective against haemopoietic cells [7,8,17–19], with the addition of a single concentration of VPA (0.6 mM) that is clinically safe and effective as an anti-convulsant [5]. Combinatorial treatments with BEZ, MPA and VPA (V-BAP) inhibited the growth of SAOS2 and MG63 cells to a greater extent than single treatments with each drug alone, in a BaP concentration-dependent manner, whilst still having much less effect on the growth of human MSCs. For the V-BAP drug combination, the concentrations of VPA and MPA that were effective in inhibiting SAOS2 and MG63 cell proliferation, i.e., 0.6 mM VPA and up to 10 μM MPA, respectively, are very likely to be achievable in humans, although the detailed pharmacokinetics for combination therapies have not been performed. In paediatric Burkitt’s lymphoma, treatment of patients with BEZ at levels that are likely equivalent to those concentrations of <0.5 mM BEZ that were used in this *in vitro* study, i.e., at 400 mg per day, also were well tolerated [9]. However, higher doses of BEZ can be problematic, particularly in older patients with poor kidney function, as was seen more recently in a BaP dose escalation study [20]. Therefore, the development of V-BAP as a potential therapeutic for patients with osteosarcoma would require careful examination of drug tolerance and dose escalation and monitoring of creatinine kinase levels and estimated glomerular filtration rate.

Live cell imaging and lineage tracking experiments demonstrated that in control conditions, human MSCs were relatively slow to proliferate, but remained viable throughout the culture period and exhibitory a migratory phenotype. As expected, SAOS2 and MG63 osteosarcoma cells in control conditions proliferated more rapidly, also remaining viable throughout the culture period. MG63 cells exhibited a similar migratory phenotype to human MSCs, whilst the SAOS2 cells were the least migratory – with both a relatively short trajectory distance and slow migratory speed. Following treatment with V-BAP, both the numbers of viable SAOS2 and MG63 cells dividing during the culture period and the migration of these cells were reduced. For both of the osteosarcoma cell lines, the inhibitory effects of V-BAP on viable cell division were concentration-dependent and significantly different to any effects on human MSCs, for which there was no significant effect. Cell migration was reduced for all cell types, but only significantly so for MG63 cells. Hence, the present study has demonstrated proof of concept that combined BEZ, MPA and VPA treatment has deleterious effects on the growth and migration of human osteosarcoma cells without adversely affecting non-transformed human MSCs.

The mechanisms of action of V-BAP in targeting osteosarcoma cells are likely to be complex and warrant subsequent studies. VPA has been used in clinical practice as an anticonvulsant for more than four decades [21] and at higher doses is known to function as a HDAC inhibitor [5], which can result in general hyperacetylation of histones and increased transcriptional activity through relaxation of DNA conformation [22]. Khanim et al. (2009) reported that BAP treatment of AML cells increased pools of prostaglandin D_2_ (PGD_2_) via: (i) MPA-mediated inhibition of the PGD_2_ metabolising enzyme Aldo-Keto Reductase (AKR) 1C3, and (ii) increased PGD_2_ synthesis through the isoprostane pathway, which was resultant from BEZ-induced reactive oxygen species (ROS) generation and lipid peroxidation [7]. In the absence of AKRIC3 activity (blocked by MPA) PGD2 spontaneously generates 15-deoxy Δ^12, 14^prostaglandin J_2_ (15d-PGJ_2_) [7], an endogenous ligand for peroxisome proliferator-activated receptor-γ (PPARγ). Increased activation of PPARγ by 15d-PGJ_2_ and other ligands has been associated with growth inhibition and increased apoptotic cell death in a wide variety of malignancies [23]. For example, ROS-induced 15d-PGJ_2_ synthesis in SAOS2, MG63 and U2 OS osteosarcoma cells, resulted in cell cycle inhibition [11]. Treatment of chondrosarcoma cells with the selective PPARγ ligand pioglitazone or 15d-PGJ_2_ also inhibited their proliferation and induced apoptosis in a dose-dependent manner [24]. The action of 15d-PGJ_2_ was more potent than pioglitazone that may reflect additional non-PPARγ-dependent actions of this electrophilic prostanoid that also covalently modifies nucleophiles, such as the free cysteine residues of proteins [25]. Another study identified that 15d-PGJ_2_ induced chondrosarcoma cell apoptosis is associated with down-regulation of the anti-apoptotic gene *Bcl-xL* and up-regulation of pro-apoptotic gene *Bax* [12]. Ewing’s sarcomas, again thought to derive from MSC transformation [26], are sensitive to TRAIL through binding to death receptor 5 (DR5) [27]. It is therefore important to note that in Jurkat lymphoma and prostate cancer cells, 15d-PGJ_2_ elevation was associated with PPARγ independent increases in protein levels of DR5 mediated by mRNA stabilization, potentiating TRAIL-induced apoptosis [28].

We have shown here that FASN and SCD-1 levels are much greater in SAOS2 and MG63 cells compared with normal human MSCs and these elevated levels decreased significantly following V-BAP treatment. FASN is a putative diagnostic indicator for osteosarcoma and its inhibition induces cell growth arrest, apoptosis and reduced cell migration in U2-OS osteosarcoma cells [29]. In addition, increased levels of SCD-1 are associated with a poor prognosis for patients with soft tissue sarcomas [30]. Hence, changes in ROS, PPARγ-dependent and PPARγ-independent activities of 15d-PGJ_2_, and altered monosaturated lipogenesis each represent plausible combinatorial mechanisms for the selective action of V-BAP against osteosarcoma cells when compared with non-transformed MSCs.

In summary, the present study has demonstrated that a novel drug combination, V-BAP, significantly inhibited the growth and viability of human osteosarcoma cells compared with non-transformed human MSCs. This supports the concept that drug combination and re-deployment strategies that have shown promise in the targeted treatment of one form of human cancer may well have application in the treatment of different forms of cancer.

## Supplementary Material

Supplementary Figures S1-S2

## Data Availability

Data generated and analysed during the present study are included in this published article. Data and materials are available from the corresponding author subject to reasonable request and subject to the ethical approvals in place and material transfer agreements.

## Abbreviations

BEZ: bezafibrate
MPA: medroxyprogesterone acetate
MSC: mesenchymal stem/stromal cell
PBS: phosphate-buffered saline
RIPA: radioimmunoprecipitation assay
ROI: regions of interest
V-BAP: combined valproic acid, bezafibrate and medroxyprogesterone acetate
VPA: valproic acid
