# Supplementary material for: Combined bezafibrate, medroxyprogesterone acetate and valproic acid treatment inhibits osteosarcoma cell growth without adversely affecting normal mesenchymal stem cells
Source: Biosci Rep. 2021 Jan 5;41(1):BSR20202505. doi: 10.1042/BSR20202505 (PMC7786328; doi:10.1042/BSR20202505)
Supplement: Supplementary Figures S1-S2 [file BSR-2020-2505_supp.pdf]

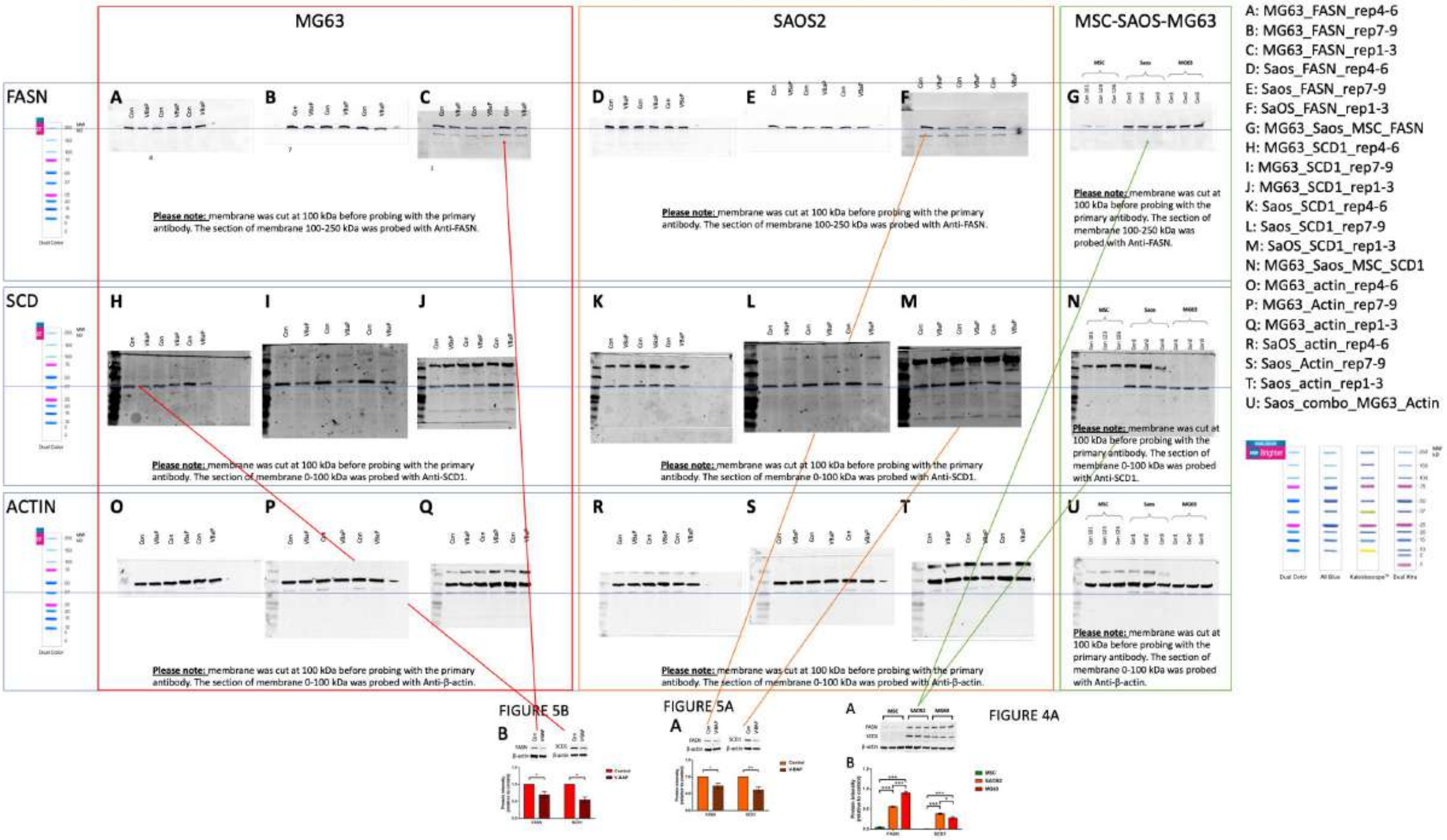

MG63
SAOS2
MSC-SAOS-MG63
FASN
SCD
ACTIN
A
B
C
D
E
F
G
H
I
J
K
L
M
N
O
P
Q
R
S
T
U
Please note: membrane was cut at 100 kDa before probing with the primary antibody. The section of membrane 100-250 kDa was probed with Anti-FASN.
Please note: membrane was cut at 100 kDa before probing with the primary antibody. The section of membrane 100-250 kDa was probed with Anti-FASN.
Please note: membrane was cut at 100 kDa before probing with the primary antibody. The section of membrane 100-250 kDa was probed with Anti-FASN.
Please note: membrane was cut at 100 kDa before probing with the primary antibody. The section of membrane 0-100 kDa was probed with Anti-SCD1.
Please note: membrane was cut at 100 kDa before probing with the primary antibody. The section of membrane 0-100 kDa was probed with Anti-SCD1.
Please note: membrane was cut at 100 kDa before probing with the primary antibody. The section of membrane 0-100 kDa was probed with Anti-SCD1.
Please note: membrane was cut at 100 kDa before probing with the primary antibody. The section of membrane 0-100 kDa was probed with Anti-β-actin.
Please note: membrane was cut at 100 kDa before probing with the primary antibody. The section of membrane 0-100 kDa was probed with Anti-β-actin.
Please note: membrane was cut at 100 kDa before probing with the primary antibody. The section of membrane 0-100 kDa was probed with Anti-β-actin.
FIGURE 5B
FIGURE 5A
FIGURE 4A
A: MG63_FASN_rep4-6
B: MG63_FASN_rep7-9
C: MG63_FASN_rep1-3
D: Saos_FASN_rep4-6
E: Saos_FASN_rep7-9
F: SaOS_FASN_rep1-3
G: MG63_Saos_MSC_FASN
H: MG63_SCD1_rep4-6
I: MG63_SCD1_rep7-9
J: MG63_SCD1_rep1-3
K: Saos_SCD1_rep4-6
L: Saos_SCD1_rep7-9
M: SaOS_SCD1_rep1-3
N: MG63_Saos_MSC_SCD1
O: MG63_actin_rep4-6
P: MG63_Actin_rep7-9
Q: MG63_actin_rep1-3
R: SaOS_actin_rep4-6
S: Saos_Actin_rep7-9
T: Saos_actin_rep1-3
U: Saos_combo_MG63_Actin

Supplementary Figure 1. All SDS-PAGE and Western blotting performed to determine FASN and SCD-1 levels in cell lines.

The original images from all experiments performed to generate data shown in Figure 4 and Figure 5 have been shown as un-cropped images, with individual single or paired (control versus VBAP treatment) lanes that were used in main Figures highlighted. Note that for all blots, the membranes were cut at 100kD with the upper section probed with antibodies for FASN or SCD-1 and the lower section (0-100kD) probed with antibodies for β–actin.

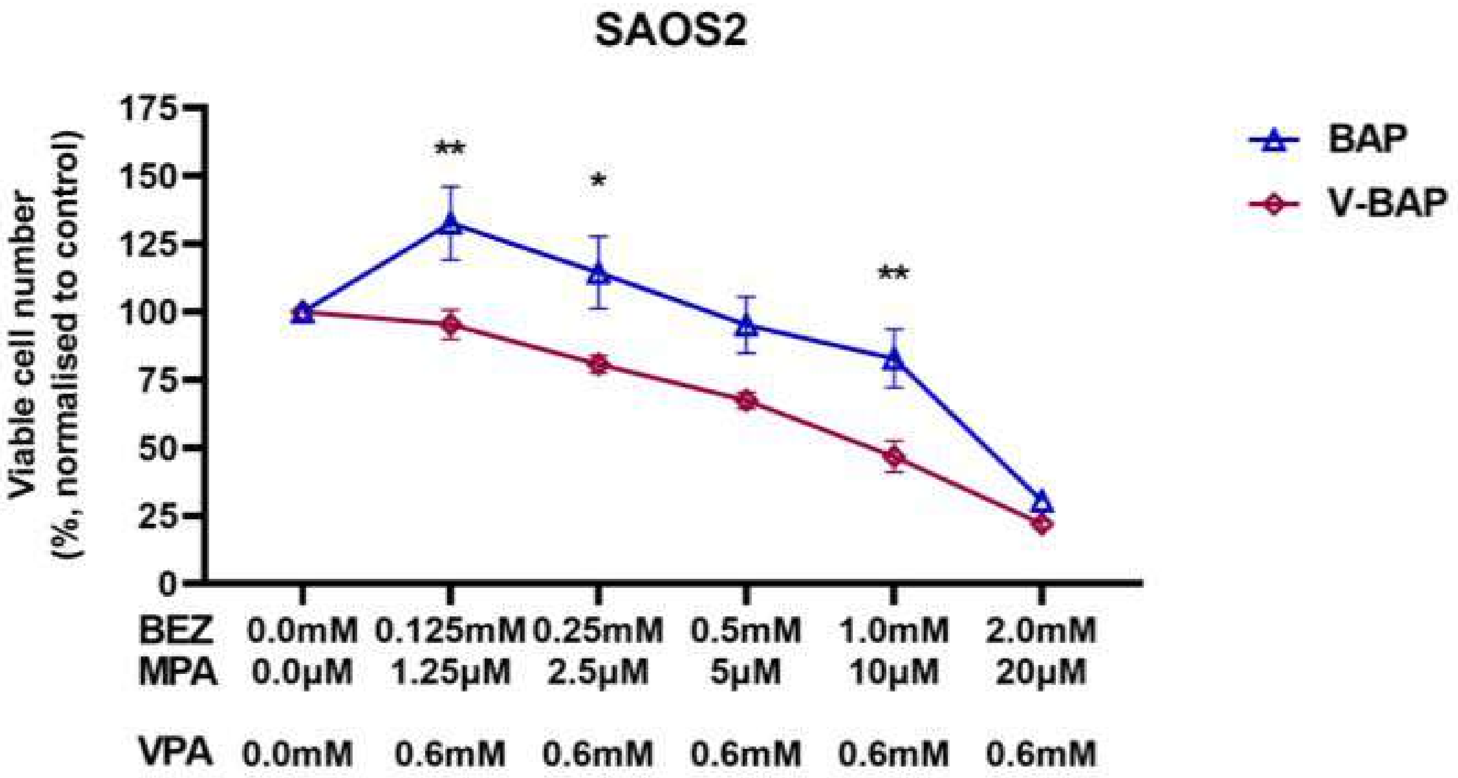
SAOS2
175
150
125
100
75
50
25
0
Viable cell number
(%, normalised to control)
**
*
**
BAP
V-BAP
BEZ 0.0mM 0.125mM 0.25mM 0.5mM 1.0mM 2.0mM
MPA 0.0μM 1.25μM 2.5μM 5μM 10μM 20μM
VPA 0.0mM 0.6mM 0.6mM 0.6mM 0.6mM 0.6mM

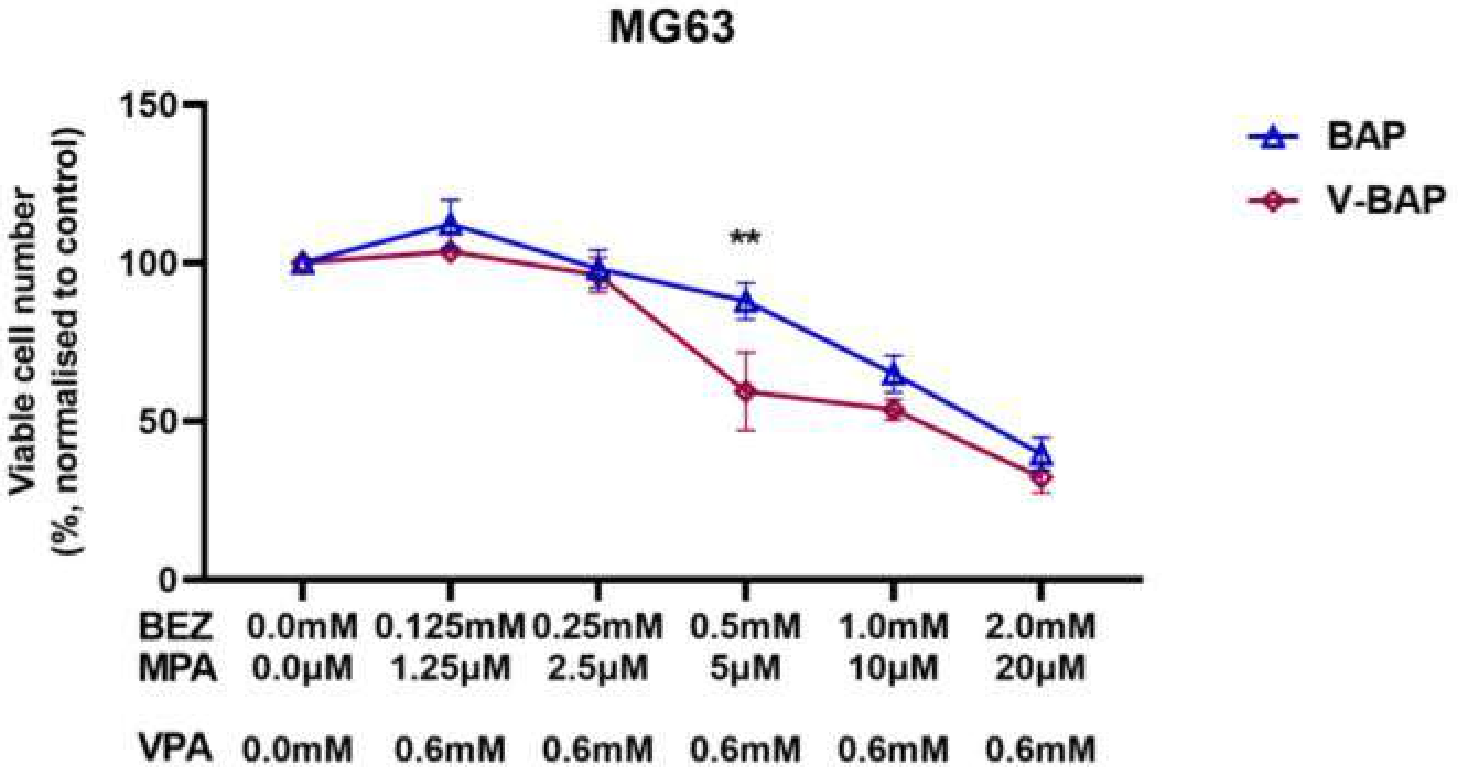
MG63
150
100
50
0
Viable cell number
(%, normalised to control)
**
BAP
V-BAP
BEZ 0.0mM 0.125mM 0.25mM 0.5mM 1.0mM 2.0mM
MPA 0.0μM 1.25μM 2.5μM 5μM 10μM 20μM
VPA 0.0mM 0.6mM 0.6mM 0.6mM 0.6mM 0.6mM

Supplementary Figure 2. V-BAP treatment more effectively inhibited SAOS2 and MG63 osteosarcoma cell proliferation than BaP treatment.

MTT assays of viable cell numbers were performed after 120 hours of culture in the presence of increasing concentrations of BEZ and MPA in V-BAP compared with BaP alone. Data shown as mean±SEM(a minimum of n=3 independent experiments) **$p<0.01$, ***$p<0.001$. Post-hoc analysis for significant differences between V-BAP versus BaPat each concentration have been indicated.
